# Structural Optimization of Magnesium Alloy Rib Claw and Evaluation of Its Mechanical Reliability In Vitro and In Vivo

**DOI:** 10.3390/ma19091833

**Published:** 2026-04-29

**Authors:** Jie Shen, Ziming Wang, Hua Huang, Zhenhua Chu, Jian Zhang, Lin Yin

**Affiliations:** 1 Department of Mechanical Engineering, College of Engineering, Shanghai Ocean University, Shanghai 201306, China; j-shen@shou.edu.cn (J.S.); wzming1113@sina.com (Z.W.);; 2 National Engineering Research Center of Light Alloy Net Forming and State Key Laboratory of Metal Matrix Composite, Shanghai Jiao Tong University, Shanghai 200240, China; 3 Shanghai Innovation Medical Technology Co., Ltd., Shanghai 201306, China

**Keywords:** magnesium alloy rib claws, structural optimization, finite element analysis, mechanical reliability, in vivo evaluation

## Abstract

**Highlights:**

- A “design-for-degradation” approach reduces stress concentration in Mg alloy rib claws.
- Optimized Gen3 Mg claws show superior yield load (358 ± 21 N) with minimal mechanical anisotropy vs. commercial Ti claws.
- A 24-week large animal model confirms controlled degradation with maintained mechanical integrity during bone healing.
- Finite element analysis (FEA) validates a 54.15% reduction in maximum von Mises stress via structural optimization.

**Abstract:**

The structural optimization and mechanical reliability of novel biodegradable JDBM magnesium alloy rib claws were investigated in this study. Simulation analysis, in vitro bending tests, and a 24-week animal implantation experiment demonstrated the promising application potential of the optimized Gen3 magnesium alloy rib claw. Compared with the pre-optimized Gen1 design, finite element analysis (FEA) confirmed that the Gen3 claw—achieved by increasing the width from 6.0 mm to 8.52 mm and adding 1.65 mm transitional fillets at stress concentration zones—resulted in a 54.15% reduction in the maximum von Mises stress (from 116.91 MPa to 53.59 MPa) and a 54.4% decrease in the equivalent strain. In vitro four-point bending tests (ASTM F382-compliant, 11.7 mm span) showed that the Gen3 magnesium claws exhibited a significantly higher yield load (358 ± 21 N) compared with titanium claws (219 ± 16 N; *p* < 0.05, independent *t*-test). A 24-week in vivo evaluation in Bama pigs further confirmed the excellent mechanical reliability of the optimized Gen3 magnesium alloy rib claw, and no fractures were observed throughout the implantation period.

## 1. Introduction

In cases of blunt thoracic trauma, rib fractures stand out as one of the most prevalent injuries, with their incidence falling between 20% and 39%. Their high occurrence and potential for serious complications, such as respiratory failure and pneumonia, significantly affect patient outcomes [1]. Rib fractures have been established as a significant cause of death in trauma patients, with reported mortality rates of up to 20 percent [2]. Additionally, both mortality and pneumonia rates increase linearly with the number of fractured ribs; each additional fracture raises the mortality risk in elderly patients by 19% [3]. While traditional conservative treatments, like chest wall strapping and pain medications, can relieve some symptoms, they are often insufficient for multiple, displaced rib fractures, especially in flail chest cases. The loss of chest wall stability in these situations usually leads to severe complications such as respiratory dysfunction, atelectasis, pulmonary infections, and even higher mortality. Surgical Stabilization of Rib Fractures (SSRF) has been shown to significantly improve patient outcomes by restoring chest wall stability and reducing the risk of complications [4]. Multiple studies confirm that SSRF minimizes the duration of mechanical ventilation, shortens ICU stays, lowers pneumonia rates, and improves long-term quality of life [5,6]. However, the therapeutic effect of SSRF is mainly dependent on the implants used.

Currently, the rib fixation devices commonly used in clinical practice primarily consist of metal fixators (e.g., titanium alloy or stainless steel rib claws) and absorbable polymer fixators (e.g., polylactic acid (PLA), polyglycolic acid (PGA), and their copolymers). The former possess high mechanical strength and are suitable for severe flail chest, but suffer from stress shielding effects (potentially causing bone resorption or implant loosening), imaging interference, foreign body reactions, and the need for secondary removal surgery [7,8,9,10,11]. The latter can degrade, thus avoiding the problem of secondary surgery; however, its mechanical properties are relatively low; reported yield loads for such polymer rib fixation devices are typically below 100 N in comparable bending tests, which limits their wide application in load-bearing rib fractures [12]. The limitations of these materials have driven the development of biodegradable metallic materials. Magnesium alloys are considered ideal candidates for bone fracture repair due to their elastic modulus matching that of human bone tissue, degradation products (Mg^2+^ ions) participating in bone metabolism and promoting bone healing, alongside good biocompatibility and biodegradability [7,11,13]. Many biodegradable medical implants are based on magnesium alloys. In orthopedics, various biodegradable magnesium-based implants have been developed for bone repair and fracture fixation, including porous magnesium alloy scaffolds, resorbable magnesium wires, magnesium-based alloy skull repair (MASR), bone plates, screws, and nails. Therefore, the development of magnesium alloy rib fixation devices is very likely to have promising clinical application prospects and benefits [14,15,16,17,18,19,20].

The rib claw is one of the most commonly used devices for internal fixation of rib fractures that mimics the curved anatomical structure of the rib. Its core structure is the clamping section, typically composed of 4 pairs of claw feet. During fixation, the claw feet envelop the fractured ends of the rib. Operation with specialized instruments closes the claw feet to achieve tight apposition to the rib surface, thereby enabling stable fixation of the fracture site [4,21]. However, the application of magnesium alloys in rib fixation still faces many challenges. The decline in mechanical properties caused by degradation must be well-matched with the healing process of rib fractures. Suppose the structure of the rib claws remains unchanged, and only their manufacturing material is replaced, from titanium alloy or stainless steel to magnesium alloy. In that case, this will lead to fracture failure during their service process. This is because the mechanical properties of magnesium alloys themselves are usually lower than those of titanium alloys. In contrast, the degradation of magnesium alloys will further lead to a decline in their mechanical properties. Therefore, to realize the clinical application of the magnesium alloy rib claw, corresponding structural optimizations and other improvements remain necessary.

Addressing the limitations of traditional treatment methods and existing fixation materials, this study aims to develop new magnesium alloy rib claws, optimize their structure, and assess their mechanical reliability both in vitro and in vivo. While magnesium alloys have been extensively studied for bone plates and screws, the specific geometric optimization of curved, claw-like rib fixation devices to mitigate degradation-induced stress corrosion cracking remains largely unexplored. Therefore, this study presents for the first time a topology-optimized JDBM magnesium alloy rib claw (Gen3) that achieves >50% reduction in peak von Mises stress via a dual strategy of main body widening and transitional fillet addition, and validates its in vivo mechanical integrity over 24 weeks in a large animal model, offering a clinically viable, degradation-matched alternative to permanent titanium implants. The present work introduces a validated ‘design-for-degradation’ topological approach distinct from simple material substitution.

## 2. Materials and Methods

### 2.1. Materials and Claws Manufacturing

The magnesium alloy rib claw implants and corresponding preformed sheets used in this study were supplied by Shanghai Innovation Medical Technology Co., Ltd. These devices are made of a magnesium alloy designated as JDBM, with a nominal composition of Mg–2.8Nd–0.2Zn–0.4Zr. In this alloy system, Nd contributes to the formation of a stable rare-earth oxide film that enhances corrosion resistance. Meanwhile, the trace additions of Zn and Zr aid in grain refinement, thereby optimizing the alloy’s corrosion degradation behavior and mechanical properties [22,23,24,25]. Compared to the commercial AZ31 magnesium alloy, JDBM exhibits a lower tendency for pitting corrosion and a more uniform degradation mode. The JDBM alloy was prepared via hot extrusion, which results in fine grains and a characteristic microstructure consisting of an α-Mg matrix with Mg_12_Nd second-phase particles distributed along the grain boundaries. This microstructure is highly effective in inhibiting localized corrosion. Regarding mechanical performance, JDBM exhibits an optimal balance of strength and plasticity. This combination provides adequate mechanical support in the early stages of implantation while maintaining structural integrity throughout the degradation process. Owing to its excellent corrosion resistance, uniform degradation, good biocompatibility, and suitable mechanical properties, the JDBM alloy system is not only applicable for vascular stents and orthopedic internal fixation implants but also serves as an excellent candidate for the rib claw implants and preformed sheets investigated in this study [26,27,28].

Since the mechanical properties of the JDBM magnesium alloys are lower than those of titanium alloys, the sheets used for manufacturing rib claws should be slightly thicker. Specifically, in this study, the titanium alloy rib claws in the control group had a thickness of 1.0 mm, while magnesium alloy sheets with a thickness of 1.1 ± 0.02 mm were selected. The optical microstructure of the sheets was characterized using an optical microscope (OM). The samples for OM observation were prepared through sectioning, grinding, polishing, and final etching. Similarly, the sample for X-ray diffraction (XRD) analysis was a section cut from the final rib claw, with its surface mechanically polished to ensure a flat, stress-free condition for accurate measurement. This sheet was first subjected to laser cutting, then to stamping at ambient temperature, and subsequently annealed at 500 °C for 10 min. It was also subjected to two-pass mechanical polishing using abrasive particles with a size of 2–3 μm for 20 min per pass, resulting in the rib claw samples required for the experiment.

### 2.2. Structural Design of Magnesium Alloy Rib Claws

**Figure 1:**
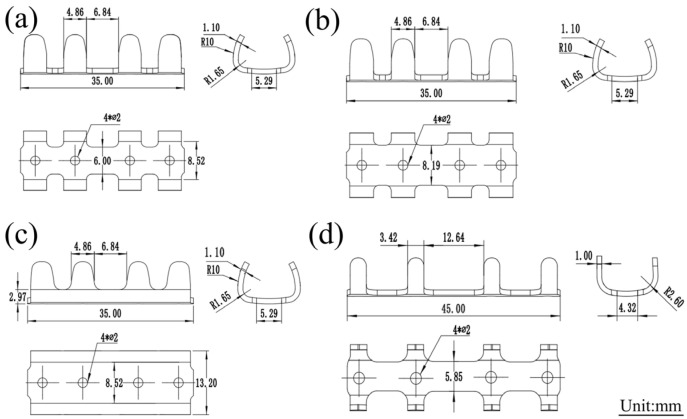
(**a**) Gen 1 Magnesium Alloy Rib Claws; (**b**) Gen 2 Magnesium Alloy Rib Claws; (**c**) Gen 3 Magnesium Alloy Rib Claws; (**d**) Titanium alloy rib claw.

Three iterative designs of the rib claw (labeled as Gen1, Gen2, and Gen3) were developed, as shown in Figure 1a–c. The main geometric changes were: (1) increasing the main body width from 6 mm in the original Gen 1 design to 8.52 mm in the optimized Gen2 design; and (2) adding a 1.65 mm transition fillet radius at the junction between the claw body and the main body from Gen2 to Gen3. The initial width of 6.0 mm was adopted from clinically mature titanium rib claws and determined according to the anatomical cross-section of Bama pig ribs. However, the Gen1 design with 6.0 mm width suffered fracture at the middle section during implantation, and preliminary finite element analysis revealed severe stress concentration. Therefore, the width was increased to 8.52 mm in the Gen2 design as a primary structural modification to enhance mechanical strength and resist fracture. The 1.65 mm transition fillet was further introduced in Gen3 to alleviate stress concentration at the junction, as suggested by finite element analysis.

These changes aimed to reduce stress concentrations and improve structural integrity. It is noteworthy that all generational designs of the magnesium alloy rib claw (Gen1 to Gen3) were fabricated from the same JDBM alloy (Mg-2.8Nd-0.2Zn-0.4Zr), ensuring consistent material composition. Therefore, the observed improvement in mechanical performance is attributed entirely to the structural topology, namely, the increase in the main body width and the introduction of transitional fillets. For mechanical performance comparison, titanium alloy rib claws (made by Changzhou Huasen Medical Instrument Co., Ltd., Changzhou, China) were used as a reference control. The structural and dimensional data are presented in Figure 1d.

### 2.3. Finite Element Analysis (FEA) of Magnesium Alloy Rib Claw Implants

**Figure 2:**
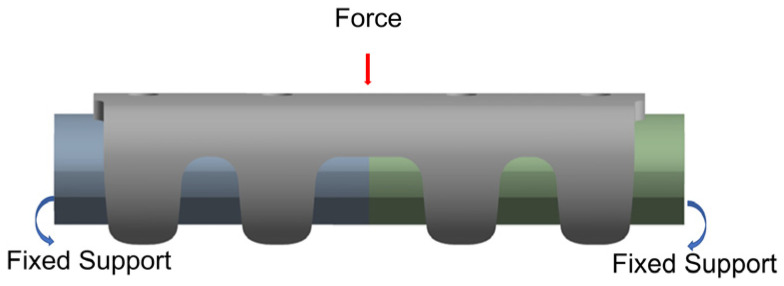
Boundary conditions and loading schematic.

Based on the SOLIDWORKS 2024 software, geometric models of a magnesium alloy rib claw and a synthetic rib were constructed. Geometry of the synthetic rib was reconstructed from CT image data of Bama pig ribs, featuring a total length of 40 mm with an approximately elliptical cross-section (central axis: 10.08 mm, minor axis: 5.30 mm). The assembled model was imported into ANSYS 2021 R1 finite element analysis software, where material properties [29,30,31] (detailed in Table 1) were assigned to both components, and meshing operations were performed (mesh parameters specified in Table 2). A bonded contact was defined between the inner surface of the rib claw and the outer surface of the rib. Boundary conditions included complete constraint fixation at both ends of the synthetic rib model, with a concentrated downward load applied at the center of the rib claw body (Figure 2). The load magnitude (46.7 N) was selected based on a previous study about thoracic loading [32].

A simplified three-point bending simulation was employed for the FEA. This approach was selected for its computational efficiency, which was crucial for the rapid iterative comparison of multiple design variants (Gen1–Gen3). Moreover, the three-point bending setup effectively accentuates stress concentration phenomena in critical regions (e.g., the mid-section and transition zones), providing a highly sensitive metric for evaluating the impact of geometric optimizations.

Although FEA utilized three-point bending, the mechanical performance conclusions were rigorously validated experimentally using ASTM F382 [33]-compliant four-point bending tests. The four-point bending method generates a uniform bending moment region. It is considered the gold standard for evaluating bone plates, thereby ensuring the biological relevance and engineering reliability of the results. This combined strategy strikes a balance between computational efficiency and experimental rigor. Finally, stress–strain distributions and structural deformation patterns were systematically analyzed using the ANSYS 2021 R1 platform, allowing for a comprehensive assessment of mechanical behavior under various design parameters.

Several simplifying assumptions were made in the computational model to facilitate iterative design comparisons. First, a bonded contact condition was enforced between the claw inner surface and the synthetic rib, which simulates the worst-case scenario of full osseointegration or friction lock but does not capture the potential micro-motion or fluid film lubrication present in vivo during early healing. Second, the simplified three-point bending load case (46.7 N) was utilized as a proxy for physiological loading; while this setup effectively isolates the critical mid-span stress concentration for comparative evaluation, it does not fully replicate the distributed, dynamic forces exerted by respiratory muscles and intrathoracic pressure changes. Despite these limitations, the comparative reduction in stress between Gen1, Gen2, and Gen3 remains valid for assessing relative structural improvements. Additional limitations pertaining to material constitutive modeling, degradation coupling, and fatigue considerations are addressed comprehensively in the (Section 4.3).

### 2.4. In Vitro Bending Mechanical Test

**Figure 3:**
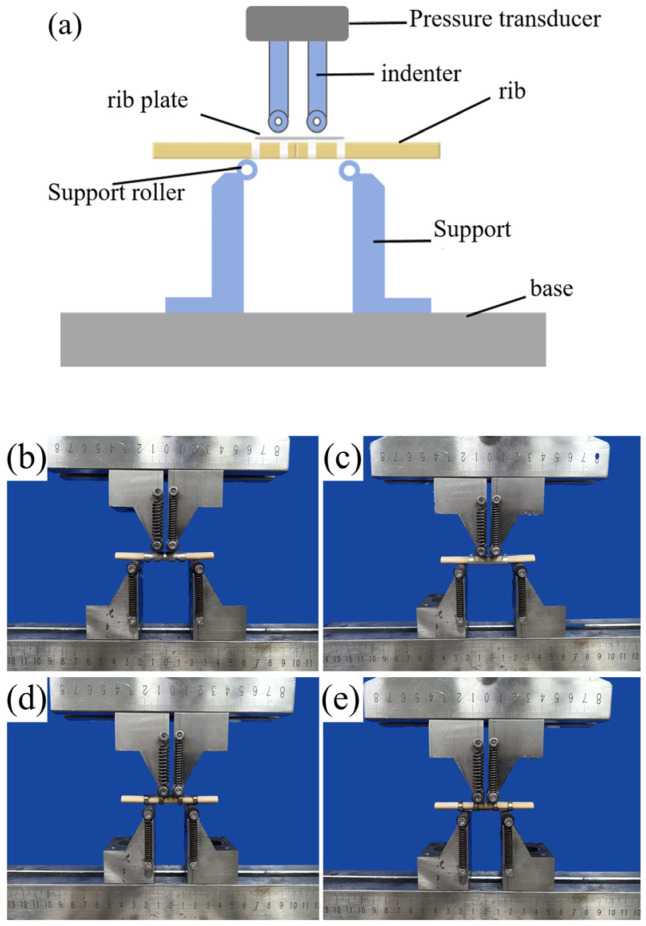
(**a**) Schematic diagram; (**b**,**c**) Magnesium alloy rib claws; (**d**,**e**) Titanium alloy rib claws.

A customized in vitro equivalent four-point bending test method, utilizing rollers with a diameter of 10 mm, was employed with non-standard fixtures designed according to the fundamental principles of ASTM F382 (Standard Specification and Test Method for Metallic Bone Plates) [33], a schematic diagram is shown in Figure 3a. The test was conducted under controlled conditions (25 ± 5 °C, RH 40 ± 10%) using an AGS-X-300kN electronic universal testing machine (SN: ORE-006) Shanghai Aose Testing Technology Co., Ltd. (Orthotek Lab), Shanghai, 200072, China. Artificial bone blocks replicating the post-bending cross-section of rib claws (10.08 mm length × 5.30 mm width) were machined to a total length of 40 mm.

Static bending tests on magnesium alloy (Figure 3b,c) and titanium alloy rib claws (Figure 3d,e) were performed under displacement control at 5 mm/min, with specimen orientations divided into claw inner surface downward (Figure 3b,d) and upward (Figure 3c,e) groups. All specimens were tested on a four-point bending fixture, maintaining the ASTM-recommended 1:3 span ratio (upper loading span: 11.7 mm; lower support span: 35 mm). Three specimens were tested per configuration.

### 2.5. Animal Experiment

Healthy adult Bama pigs, weighing 60–65 kg, were randomly divided into three groups with three pigs each. The experimental group was implanted with optimized third-generation (Gen3) magnesium alloy rib claws. In contrast, the control group received both first-generation (Gen1) magnesium alloy rib claws and commercially available titanium alloy rib claws. Under general anesthesia, a standardized complete transverse rib fracture model was created through left thoracotomy: the 9th–10th intercostal space was exposed, and a midshaft osteotomy was performed perpendicular to the rib’s long axis with an oscillating saw. Great care was taken to avoid damaging adjacent tissues during surgery, ensuring the model’s consistency. All implants underwent standardized sterilization before fracture reduction and anatomical restoration. The claw arms were secured around the fracture ends using elastic deformation-generated clamping force, ensuring bone–implant contact and fixation stability. Postoperative serial thoracic X-ray/CT imaging was performed at weeks 2, 4, 8, 12, and 24. Avizo software (Avizo 2020) was used to analyze CT data for (1) implant positional migration, (2) structural abnormalities (e.g., fractures), and (3) rib fracture healing progress. Ethical statement: All animal experiments were approved by the Institutional Animal Care and Use Committee (IACUC) of Shanghai Hansai Medical Technology Co., Ltd., Shanghai, China (approval No. IACUC-2024-114) and conducted in strict accordance with international guidelines for the care and use of laboratory animals (ARRIVE 2.0).

### 2.6. Statistical Analysis

Quantitative data are presented as mean ± standard deviation (SD). The primary endpoint was defined a priori as the comparison of yield load between Gen3 magnesium claws and titanium claws, assessed using an independent two-sample *t*-test. All other mechanical parameters (yield displacement, bending stiffness, bending strength) were analyzed descriptively and are presented without inferential statistical comparisons. A *p*-value < 0.05 was considered statistically significant.

## 3. Results

### 3.1. Microstructural Characterization of Magnesium Alloy Rib Claws

**Figure 4:**
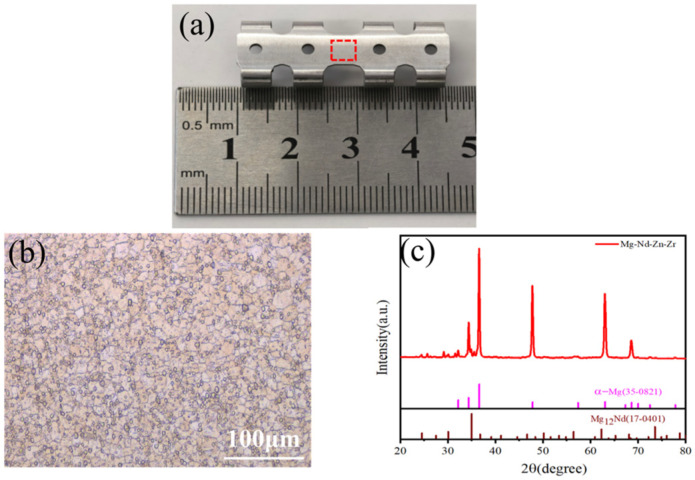
(**a**) Image of a rib claw plate made from Mg-Nd-Zn-Zr alloy. (**b**) Optical micro-structure of the sheet used for rib claw fabrication. The red box indicates the location for metallographic sampling. (**c**) XRD pattern of the sheet.

The as-fabricated magnesium alloy rib claw is shown in Figure 4a. As shown in the OM image of the sheet material in Figure 4b, the microstructure consists of uniform and fine equiaxed grains with clearly visible grain boundaries. The average grain size was estimated to be approximately 4.2–5.5 μm, as determined using image analysis software and a scale bar. The XRD pattern collected at a scanning rate of 20°/min is presented in Figure 4c, showing that the prominent diffraction peaks correspond to the α-Mg matrix phase and the Mg_12_Nd intermetallic compound phase. Furthermore, no obvious aggregation or coarse particles of secondary phases were observed in the optical micrograph.

### 3.2. Numerical Simulation Results and Validation

**Figure 5:**
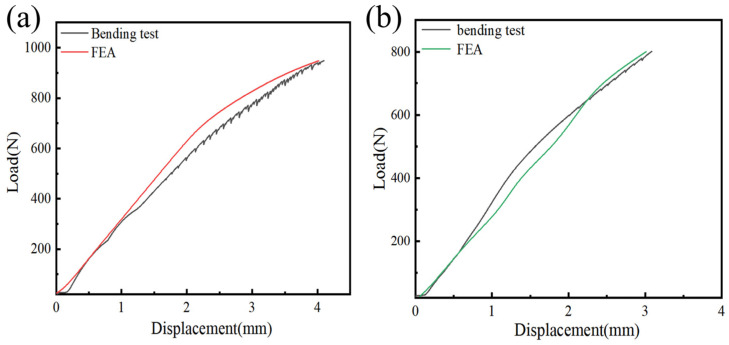
Verification and Comparison Chart of Force-Curves between Four-Point Bending Test (11.7 mm Span) and Finite Element Simulation Results for the Gen3 Magnesium Alloy Rib Claws: (**a**) Inner surface of the claw facing downward, (**b**) Inner surface of the claw facing upward.

To validate the effectiveness of the model, bending tests were performed on the Gen3 magnesium alloy rib claw under two loading configurations: with the inner surface of the clamping arm facing downward and with it facing upward. The experimentally measured force–displacement curves from the bending tests were compared with predictions from FEA. As shown in Figure 5a (inner surface facing downward) and Figure 5b (inner surface facing upward), the FEA curves for the Gen3 magnesium alloy rib claw closely matched the experimental bending curves in both configurations. Both showed a steady increase in force with increasing displacement, and the curve profiles aligned well. This demonstrated that the finite element model accurately simulated the force–displacement relationship of the Gen3 magnesium alloy rib claw during actual bending, confirming its reliability under different clamping orientations. Therefore, the validated model provided a solid basis for further analysis of the mechanical behavior of the Gen3 magnesium alloy rib claw.

Considering that the Gen1 and Gen2 magnesium alloy rib claws, although optimized in structural details such as main body width and transition fillets, were based on the same JDBM magnesium alloy substrate and used consistent material parameters (e.g., elastic modulus, Poisson’s ratio), meshing strategies (e.g., element type, mesh density), and boundary condition settings (e.g., clamping method, loading path) in the finite element modeling process, the core logic and reliability of the modeling approach were confirmed through the experimental validation of the Gen3 model. Therefore, it could be reasonably inferred that the finite element models constructed for the Gen1 and Gen2 magnesium alloy rib claws also possessed a sound modeling basis and predictive credibility, and their simulation results could provide a practical reference for analyzing the mechanical performance of the Gen1 and Gen2 rib claws.

**Figure 6:**
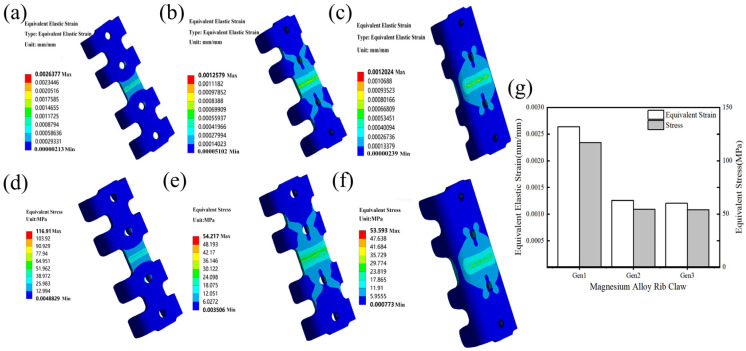
Stress–strain analysis of rib claws: (**a**–**c**) Distribution of equivalent plastic strain for Gen1, Gen2, and Gen3, respectively; (**d**–**f**) Distribution of equivalent stress for Gen1, Gen2, and Gen3 rib claws, respectively; (**g**) Comparative bar chart of the three.

Figure 6 displays the stress and strain contour maps from finite element analysis for three typical generations of rib claw structures. Quantitative FEA results show that the initial Gen1 structure had a maximum equivalent strain of 0.0026377 and a maximum equivalent stress of 116.91 MPa. Equivalent strain is a broad measure of how much the structure deforms—higher values indicate more severe local plastic deformation. After the first round of optimization, the Gen2 structure reduced the maximum equivalent strain to 0.0012579, approximately 52.3% lower than that of the Gen1 structure. Following the second optimization, the final Gen3 structure further decreased this to 0.0012024, representing a total reduction of roughly 54.4% from Gen1. This downward trend demonstrates that from Gen1 to Gen3, the structures’ overall stiffness increased, and their ability to control deformation steadily improved. Equivalent stress (Von Mises stress) is used to evaluate the risk of material failure under complex stress conditions—higher stresses suggest a greater chance of plastic deformation or failure. The Gen2 structure’s maximum equivalent stress was 54.217 MPa, which is a 53.6% reduction from Gen1. The Gen3 structure further reduced this stress to 53.593 MPa, representing a 54.15% reduction compared to Gen1, indicating significantly improved load capacity and resistance to yield.

From Gen1 to Gen3, in the crucial mid-section loading area, maximum displacement, total stress, and equivalent strain/stress all showed consistent decreasing trends. The Gen3 design represents a significant milestone in performance, with a maximum equivalent stress decrease from 116.91 MPa to 53.593 MPa (a 54.15% reduction) and a maximum equivalent strain reduction of 54.4% (from 0.0026377 to 0.0012024). This systematic optimization significantly improved the structural stiffness and yield resistance.

While full-field strain validation (e.g., via digital image correlation) was not performed due to the complex curvature and small scale of the rib claws, an important indirect validation of the stress distribution is provided by the in vivo fracture behavior of the Gen1 implants. As shown in the CT images of Gen1 in Section 3.4, all Gen1 magnesium alloy claws fractured within 4 weeks post-implantation, and the fracture consistently occurred at the junction between the claw arm and the main body. This location corresponds precisely to the region of maximum von Mises stress identified in the finite element analysis (116.91 MPa, Figure 6d). The exact correspondence between the predicted high-stress region and the observed failure site provides strong circumstantial evidence that the stress distribution captured by the FEA model is physically meaningful.

The stress and strain values reported above were obtained using a simplified three-point bending configuration, which was employed to facilitate rapid design iteration. To ensure that the comparative conclusions are not an artifact of this simplification, additional simulations were performed using a four-point bending setup that precisely replicates the experimental validation conditions (ASTM F382) [33]. Under four-point bending, the maximum von Mises stress for Gen1 was 113.02 MPa, decreasing to 57.34 MPa for Gen3, corresponding to a 49.3% reduction. The equivalent strain decreased from 0.002826 to 0.001405, a 50.3% reduction. These relative improvements are highly consistent with those obtained under three-point bending (54.2% stress reduction and 54.4% strain reduction). Furthermore, the location of peak stress remained identical (junction of claw arm and main body) in both configurations. These results confirm that while absolute stress magnitudes differ between loading modes due to the different moment distributions inherent to three-point and four-point bending, the relative ranking of the designs and the identification of the critical stress concentration region are robust to the loading simplification. Therefore, the comparative trends reported throughout this study are not artifacts of the simplified FEA boundary conditions.

### 3.3. Bending Performance of Rib Claws: Mg/Ti-Alloy Comparison

**Figure 7:**
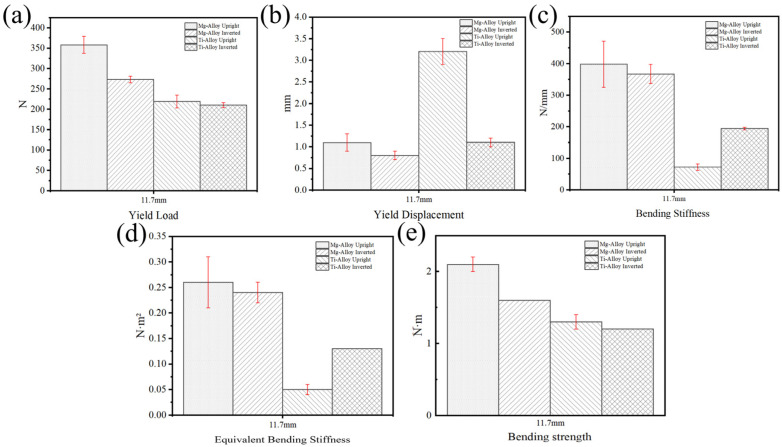
Four-point bending test of magnesium alloy and titanium alloy rib claws: (**a**) Yield Load; (**b**) Yield Displacement; (**c**) Bending Stiffness; (**d**) Equivalent Bending Stiffness; (**e**) Bending Strength.

Based on the research above, the performance of the Gen3 magnesium alloy rib claw was compared with that of the titanium alloy rib claw. As shown in Figure 7a–e, the Gen3 magnesium alloy rib claw demonstrated excellent mechanical performance in both upright and inverted configurations. The yield load for the upright configuration was (358 ± 21) N, significantly higher than that of the inverted configuration (*p* < 0.05, *t*-test) at (273 ± 8) N (Figure 7a). Regarding yield displacement (Figure 7b), the upright configuration reached (1.1 ± 0.2) mm, slightly exceeding the inverted configuration’s value of (0.8 ± 0.1) mm. Concerning bending stiffness (Figure 7c), the upright and inverted configurations displayed values of (390.8 ± 73.4) N/mm and (367.5 ± 30.3) N/mm, respectively; their equivalent bending stiffness values were also comparable (upright: 0.26 ± 0.05 N·m^2^; inverted: 0.24 ± 0.02 N·m^2^) (Figure 7d). For bending strength (Figure 7e), the upright configuration reached (2.1 ± 0.1) N·m, slightly surpassing the inverted configuration’s value of (1.6 ± 0) N·m.

In contrast, the mechanical performance of the titanium alloy rib claws was found to be highly dependent on loading configuration. As shown in Figure 7a–e, notable differences were observed in bending stiffness and yield displacement between orientations, highlighting the structural anisotropy inherent in traditional titanium designs. The yield loads for upright and inverted configurations were (219 ± 16) N and (210 ± 6) N, respectively (Figure 7a), indicating little difference in strength. However, the yield displacement in the upright configuration was (3.2 ± 0.3) mm, and in the inverted configuration it was (1.1 ± 0.1) mm (Figure 7b). Bending stiffness was measured as (71.8 ± 9.7) N/mm for upright and (194.7 ± 4.0) N/mm for inverted configurations (Figure 7c), representing an approximate 63% reduction in mean stiffness value for the upright orientation. Similarly, the equivalent bending stiffness was considerably reduced in the upright orientation (0.05 ± 0.01 N·m^2^) compared to the inverted configuration (0.13 ± 0 N·m^2^) (Figure 7d). Bending strength for the upright configuration was (1.3 ± 0.1) N·m, slightly lower than in the inverted configuration at (1.2 ± 0) N·m (Figure 7e). Notably, such marked configuration sensitivity was absent in the topologically optimized Gen3 magnesium alloy claw, which showed more consistent mechanical behavior across the two tested orientations, suggesting reduced directional sensitivity under these specific bending conditions. This consistency suggests improved predictability of performance under these quasi-static test conditions.

**Figure 8:**
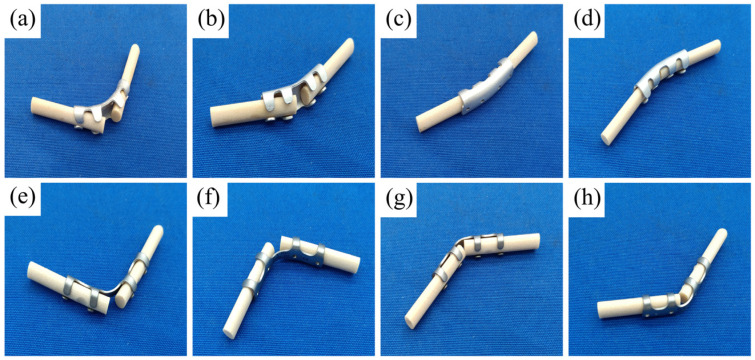
Four-point bending test of magnesium alloy and titanium alloy rib claws: (**a**–**d**) Deformation profiles of Mg alloy rib claws after load application in in vitro tests; (**e**–**h**) Deformation profiles of titanium alloy rib claws after load application in in vitro tests.

Under identical four-point bending conditions, the Gen3 magnesium alloy showed notably higher mechanical strength values compared to the titanium alloy controls in these specific bending configurations, as indicated by its higher yield load. The photos of the magnesium alloy and titanium alloy claw after bending tests were shown in Figure 8 (Figure 8a–d for magnesium alloy; Figure 8e–h for titanium alloy), which confirmed that failure in both materials occurred through permanent plastic deformation of the implant, with no evidence of brittle fracture.

### 3.4. In Vivo Biomechanical Performance and Osseous Regeneration of Optimized

**Figure 9:**
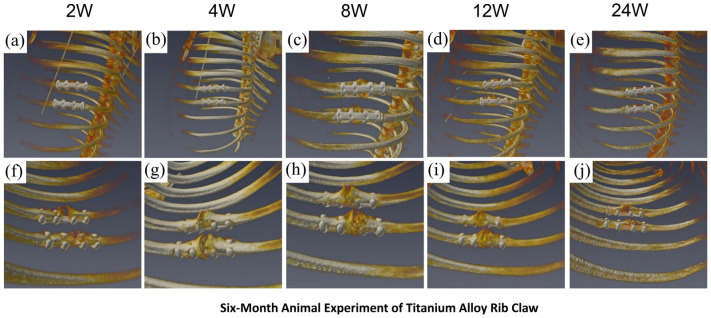
Titanium alloy claw: Stable fixation and successful healing. (**a**–**e**) Full-body CT scans reveal the stable, unchanged implant over 24 weeks. (**f**–**j**) Close-up views show continuous callus formation, leading to complete bone union.

In the titanium alloy rib claw implantation group, serial postoperative chest CT scans (weeks 2, 4, 8, 12, and 24; Figure 9a–e showing full thoracic views, Figure 9f–j showing localized fracture region views) revealed no significant displacement of the implant. Throughout the observation period, the morphology of the titanium alloy implant remained stable, with no observed fractures or other structural abnormalities, indicating excellent in vivo mechanical stability and positional maintenance. Regarding fracture healing, callus formation progressed gradually over time. Radiographic results demonstrated good bone–implant interface contact and positive integration trends, confirming that the titanium alloy rib claw provided sustained mechanical support for fracture healing.

**Figure 10:**
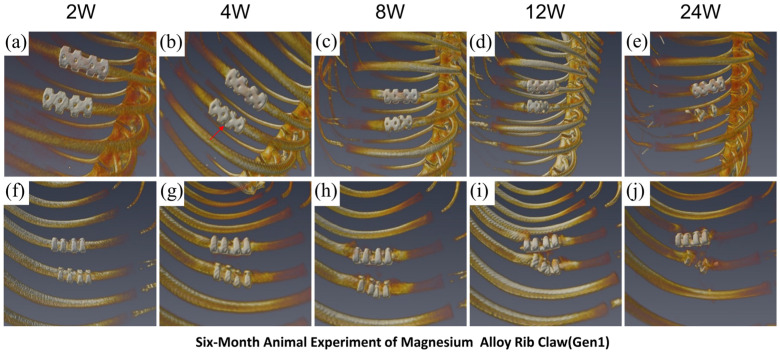
Gen1 Mg alloy claw: Early fracture and healing failure. (**a**–**e**) Full-body CT scans show the implant degrading and failing. (**f**–**j**) The claw fractures and dislodges by week 4 (red arrow), causing instability and resulting in non-union by week.

In the Gen1 magnesium alloy rib claw group, CT images (Figure 10a–e showing full thoracic views; Figure 10f–j showing localized views) revealed progressive degradation-related changes in the implant over time. Critically, in all three animals, the Gen1 implants fractured and dislodged within 4 weeks post-operation. Fractures primarily occurred in the middle section of the claw, consistent with the stress concentration area identified by FEA, resulting in catastrophic fixation failure. Subsequently, by week 8, the lack of stable mechanical support impeded callus formation. By week 24, radiographic assessment confirmed incomplete callus remodeling and failure to achieve complete fracture healing in all cases, which is directly attributed to the premature structural failure of the implants. These in vivo results are in perfect agreement with FEA predictions, which indicated a high stress concentration (maximum von Mises stress of 116.91 MPa) and strain in the Gen1 design. Its inferior mechanical performance led to early mechanical failure, thereby disrupting the normal bone healing process.

**Figure 11:**
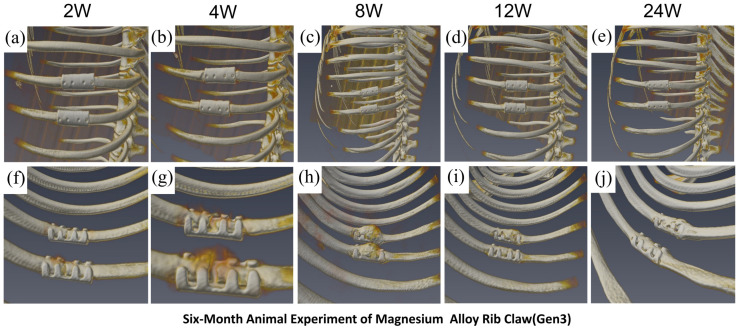
Optimized Gen3 Mg alloy claw: Successful healing with controlled degradation. (**a**–**e**) The implant shows gradual degradation but remains intact and visible for 24 weeks. (**f**–**j**) The healing process progresses normally from callus formation to complete bony union, matched by the implant’s degradation.

In the optimized Gen3 magnesium alloy rib claw group, serial CT images (Figure 11a–e showing full thoracic views; Figure 11f–j showing localized views) demonstrated in vivo characteristics different from those of the titanium alloy. The Gen3 implant showed progressive morphological changes consistent with its degradation process, while fracture healing followed dynamic developmental stages: early healing signs appeared at week 2; localized bone resorption was observed at the claw-arm contact zones by week 4; significant callus formation and improved healing occurred by week 8; callus volume began to decrease by week 12, indicating the start of remodeling, although bone resorption at the claw-arm contact zones persisted; by week 24, callus remodeling mainly was complete, resulting in bony union. Notably, the main structure of the Gen3 implant remained clearly visible at 24 weeks. FEA results showed that, through structural optimization, the maximum von Mises stress in Gen3 decreased to 53.59 MPa (a 54.15% reduction compared to Gen1), and the equivalent strain was reduced by 54.4%. This significant improvement in mechanical performance directly enhanced in vivo performance: Gen3 successfully endured physiological loads without fracture throughout the 24-week experimental period, providing continuous mechanical support for fracture healing, while its degradation behavior was well-matched to the healing process. The in vivo animal study strongly validated the accuracy of the FEA predictions, demonstrating that reducing stress concentration through topological optimization can significantly improve the in vivo mechanical performance and biomedical functionality of magnesium alloy implants.

## 4. Discussion

### 4.1. The Biomechanical Theoretical Framework for Structural Optimization

Unlike previously reported magnesium-based bone plates or hemostatic clips, rib claws experience a unique combination of bending and clamping forces around a curved cortical surface. The structural optimization reported herein specifically addresses the interplay between cross-sectional widening and fillet radius addition to counteract the stress corrosion cracking susceptibility inherent to the JDBM alloy in this particular loading scenario.

The strain data clearly show a trend: the maximum strains for Gen1, Gen2, and Gen3 are 0.0026377, 0.0012579, and 0.0012024, respectively. Since the material remains within the elastic range, the strain decreases as stress decreases. This indicates that increasing the cross-section effectively enhances structural stiffness. The fracture failure of the early-generation magnesium alloy rib claw (Gen1) mainly results from the combined effects of inadequate cross-sectional bending resistance and stress concentration at the right-angle transition areas. Using the “dual-stage mechanical optimization strategy” and incorporating finite element stress and strain data, the effectiveness of this approach in enhancing structural strength and stability was systematically confirmed.

Main body widening for nominal stress reduction [34].

(1) $$I=\frac{{bh}^{3}}{12}$$

where “*b*” represents the width and “*h*” denotes the thickness (*h* = 1.1 mm in this design). The width increased from 6.0 mm (1st Gen) to 8.52 mm (3rd Gen), resulting in an improved moment of inertia.

(2) $$\frac{I_{3rd}}{I_{1st}}=\frac{b_{3rd}}{b_{1st}}=\frac{8.52}{6.0}=1.42$$

Bending stress is inversely proportional to the moment of inertia (The bending stress σᵦ is proportional to the bending moment *M* and inversely proportional to the moment of inertia *I*, where *Y* is the distance from the neutral axis.)

(3) $$\sigma_{b}=\frac{MY}{I}$$

(4) $$\varepsilon =\frac{\sigma }{E}$$

Stiffness and reduces overall deformation.

Fillet design in transition zones suppresses local stress peaks.

According to the theory of elastic stress concentration, Pilkey’s Stress Concentration Factors Handbook [35] indicates that the theoretical elastic stress concentration factor (*Kt*) for right-angle transition structures is:

(5) $$K_{t}=1+2\sqrt{\frac{t}{r}}$$

Gen1: Employs a right-angle transition design with a theoretical stress concentration factor $k_{t1}$ = 2.5.

Gen3: Incorporates a fillet with “r” equal to 1.65 mm, reducing the theoretical stress concentration factor $K_{t3}$ = 1.2.

To quantify the independent contribution of the fillet design, a 2nd Gen control group (widened but unfilleted) was established. FEA results are as follows:

2nd Gen: Local peak stress = 54.21 MPa, local strain = 0.0012579

3rd Gen: Local peak stress = 53.59 MPa, local strain = 0.0012024

Despite having identical moments of inertia, the Gen3 design achieves an additional 1.1% reduction in peak stress and a 4.4% decrease in local strain through fillet optimization. This shows that the fillet structure effectively reduces stress concentration, smooths out local stress and strain distribution, and maximizes the overall mechanical performance of the design.

### 4.2. Advantages, Challenges, and Future Directions of the Optimized Magnesium Alloy Rib Claw

The failure mode of the Gen1 magnesium alloy claws clearly highlights the challenges faced by magnesium alloy implants in terms of stress concentration and degradation. As shown in Figure 10, the Gen1 claw effectively maintained fixation at the second week after implantation; however, a fracture occurred by the fourth week. FEA results indicated significant stress concentration in the Gen1 design, with the maximum equivalent stress reaching 116.91 MPa (Figure 6d). In a bodily fluid environment, this high-stress region is prone to stress corrosion, leading to localized corrosion rates much higher than those in other regions, thus accelerating the initiation and propagation of cracks [36]. Additionally, the degradation process of magnesium alloys causes a gradual decline in their mechanical properties. While the initial mechanical properties (although close to those of titanium alloys) barely meet fixation requirements, as degradation progresses, the residual strength in high-stress concentration areas falls below the critical value, eventually leading to structural failure [37,38].

In contrast to Gen1, the optimized Gen3 magnesium alloy claws significantly improved mechanical performance and stress distribution by widening the main body (to 8.52 mm) and adding a transition radius (r = 1.65 mm). The maximum equivalent stress decreased by 54.15%, lowering to 53.59 MPa (Figure 6f). This design not only significantly increased the initial yield load of Gen3 (358 ± 21 N), surpassing that of the titanium alloy control group (219 ± 16 N) (Figure 7a), Notably, this value is also substantially higher than the reported mechanical performance of absorbable polymer rib fixators, which typically yield at only 13.8–28.3 N in three-point bending tests [39], but also fundamentally improved stress distribution and reduced the risk of stress corrosion. Therefore, the Gen3 claw maintained its structural integrity throughout the 24-week animal experiment period, successfully avoided early fracture, demonstrated excellent mechanical reliability, and fully met the stability requirements for clinical applications.

Although Gen3 achieved significant breakthroughs in mechanical performance, localized bone resorption was observed at the claw-bone contact interface in this study (Figure 11f–j). We hypothesize two potential, non-mutually exclusive mechanisms underlying this observation. First, a mechanical origin: the elastic modulus of JDBM alloy is still higher than that of cortical bone (~10–20 GPa). Despite structural optimization, this residual stiffness mismatch may cause mild stress shielding in the peri-implant zone, leading to localized disuse osteopenia. Second, a biochemical origin: magnesium degradation releases Mg^2+^, OH^−^, and hydrogen gas locally. While moderate Mg^2+^ promotes osteogenesis [40], transient local alkalization and hydrogen evolution can activate osteoclasts and suppress osteoblasts at the interface. Importantly, the preserved mechanical integrity of Gen3 ensures that this mild resorption does not compromise fixation stability, which is a key difference from the catastrophic failure of Gen1 [40,41,42,43,44,45].

These mechanisms may act independently or synergistically, and therefore, more systematic histological and mechanical testing is required for further validation. It is also important to recognize that the translation from in vitro mechanical performance to in vivo success involves biological variables that are not captured in benchtop testing. Factors such as inter-animal variability in bone quality, soft tissue interposition, local vascularity, and individual differences in respiratory mechanics can all modulate the effective load transmitted to the implant and the local degradation microenvironment. The consistent maintenance of fixation in all three Gen3-implanted animals, despite these inherent biological variabilities, underscores the robustness of the optimized design. Nevertheless, clinical translation will require careful consideration of patient-specific anatomical and physiological factors that may influence implant performance. Future optimization should focus on the following aspects: First, surface modification techniques such as micro-arc oxidation, calcium phosphate coatings, or organic-inorganic composite coatings could be employed on the magnesium alloy materials to reduce the initial corrosion rate and prevent excessive accumulation of Mg^2+^ and hydrogen gas. Second, further optimizing the geometry of the claw-bone contact interface to increase the contact area, reduce stress concentration, and appropriately control tightening force during surgery to minimize micro-motion at the interface. Third, dynamic monitoring of local pH, inflammatory factors, and bone metabolism markers should be strengthened during animal experiments and preclinical studies, particularly during the postoperative 4–8 weeks, to clarify the specific mechanisms of bone resorption [42,46,47].

Furthermore, certain limitations in the current animal experimental design should be acknowledged and addressed in future work. While the Bama pig model provided a valuable platform for the in vivo evaluation of implant performance, its thoracic anatomy and respiratory biomechanics differ from those of humans. Additionally, the sample size of three animals per group (n = 3), while sufficient to demonstrate clear trends such as the early fracture of Gen1 implants, is relatively small. To enhance the statistical power and generalizability of the findings, subsequent confirmatory studies should employ animal models that more closely replicate human biomechanics and incorporate larger cohort sizes.

Additionally, while radiographic evidence confirmed successful bony union in both the titanium control and the optimized Gen3 magnesium alloy groups, the present study did not evaluate the post-healing mechanical properties of the repaired ribs. Future work should include ex vivo biomechanical testing (e.g., three- or four-point bending tests of the explanted, healed rib segments) to quantitatively assess whether the restored bone possesses sufficient strength and stiffness to withstand physiological loads during normal activity.

In summary, the optimized Gen3 magnesium alloy rib fixation claw did not fracture within 24 weeks, confirming its excellent mechanical reliability and ability to meet the clinical stability requirements. Although the localized bone resorption phenomenon reveals room for improvement in the “mechanical-degradation-biocompatibility” balance, the overall results demonstrate the tremendous potential and application feasibility of Gen3 claws as biodegradable implants.

### 4.3. Study Limitations and Future Directions

While the present study demonstrates the successful geometric optimization and preclinical validation of the Gen3 magnesium alloy rib claw, several limitations inherent to the modeling and experimental design must be acknowledged to guide future investigations.

First, regarding the finite element framework, the material behavior of the JDBM alloy was modeled as linear elastic (E = 42 GPa, ν = 0.35). This simplification, while computationally efficient for iterative design comparison, neglects the elasto-plastic transition, strain hardening, and—most critically—the time-dependent degradation of mechanical properties (e.g., effective elastic modulus and yield strength reduction) caused by corrosion in physiological fluid. A coupled chemo-mechanical constitutive model that explicitly links corrosion pit evolution to local stress intensification and hydrogen embrittlement is absent from the current analysis. The in vivo fracture of the Gen1 implant at precisely the location of peak elastic von Mises stress (Figure 10) provides strong circumstantial evidence that elastic stress concentration drives failure initiation, but a more sophisticated damage evolution framework would be required to predict the time-to-failure or residual load-bearing capacity during degradation.

Second, the validation of the numerical model was limited to a comparison of force-displacement curves. While the agreement between simulation and experiment for the Gen3 configuration (Figure 5) supports the credibility of the modeling approach, full-field strain validation (e.g., via digital image correlation) was not performed due to the complex curvature and small scale of the rib claws. Furthermore, the extrapolation of model validity from Gen3 to Gen1/Gen2 relies on the consistency of the meshing and boundary condition strategies, yet direct experimental validation of the force-displacement responses of the earlier designs would provide additional confidence in the absolute predicted stress values. Nevertheless, as demonstrated in Section 3.2, a sensitivity analysis comparing three-point and four-point bending simulations confirmed that the relative stress and strain reductions from Gen1 to Gen3 are consistent across loading configurations (approximately 50% in both cases), with identical peak stress locations, indicating that the comparative conclusions of this study are robust to the simplified loading conditions employed in the FEA. Additionally, the FEA model employed a bonded contact condition that neglects interfacial micro-motion and frictional effects; future work should incorporate more realistic contact formulations with time-dependent degradation.

Third, the mechanical testing and in vivo evaluation were limited in scope. The sample size (n = 3 per group) was constrained by the logistical and ethical considerations of the large-animal model. While this sample size was sufficient to capture the binary outcome of mechanical integrity (Gen3 remained intact while Gen1 fractured catastrophically), it precludes robust statistical analysis of secondary outcomes such as callus volume quantification or degradation rate variance. Additionally, this study did not evaluate the fatigue behavior of the implants under cyclic respiratory loading. Rib fixation devices are subjected to approximately 15–20 million loading cycles over a 24-week healing period. The combined effects of cyclic stress and concurrent corrosion may initiate fatigue cracks that are not predicted by static bending tests alone. Dedicated in vitro corrosion-fatigue testing in simulated body fluid is therefore an essential next step.

Fourth, it is important to clarify the nature of the design evolution presented in this work. The progression from Gen1 to Gen3 was guided by fundamental mechanics principles, as detailed in Section 4.1: increasing the main body width enhances the section modulus and reduces global bending stress, while the addition of a transition fillet mitigates stress concentration at the critical junction. These modifications represent a rational, theory-driven iterative improvement. Future work should employ rigorous multi-objective optimization methods—incorporating constraints on implant mass, surgical profile, and degradation uniformity—to identify an optimal design for clinical translation.

Finally, the degradation analysis remained qualitative. Quantitative metrics such as in vivo corrosion rate (mm/year), hydrogen gas volume evolution, and local pH fluctuation were not measured. Future work should incorporate micro-computed tomography volumetry of explanted devices and in situ electrochemical monitoring to establish the precise kinetics of degradation and its correlation with the observed peri-implant bone resorption.

In summary, while the optimized Gen3 design represents a significant advancement in addressing the stress-corrosion failure mode of magnesium alloy rib claws, the limitations outlined above underscore the need for continued refinement in both computational modeling fidelity and experimental characterization before clinical deployment.

## 5. Conclusions

This study demonstrates that topological optimization—by widening the main body to 8.52 mm and incorporating a 1.65 mm transition fillet—is a highly effective strategy for enhancing the mechanical reliability of biodegradable magnesium alloy rib claws. The optimized third-generation (Gen3) claw exhibited a 54.15% reduction in maximum von Mises stress in silico and a significantly higher yield load in vitro compared to both its predecessors and commercial titanium alloy controls. Crucially, a 24-week in vivo evaluation confirmed that the Gen3 implant maintained structural integrity without fracture, while its progressive degradation profile harmonized with the stages of bone callus formation and remodeling, achieving a critical balance between temporary mechanical support and timely biodegradation. These findings provide robust preclinical evidence that structurally optimized magnesium alloy implants can successfully bridge the gap between initial strength requirements and long-term biosorption, offering a promising alternative to permanent implants by eliminating the need for secondary removal surgery. Future research should focus on elucidating the bone–implant interface biology under dynamic loading and advancing towards patient-specific designs.

It should be noted that clinical application ultimately requires implants to be equally reliable under long-term dynamic cyclic loading. Therefore, future research will focus on validating the long-term durability of this optimized design under fatigue loading conditions that more closely mimic physiology and on investigating the coupling between its degradation and mechanical properties in a dynamic environment. This will complete the translation from proof-of-concept to comprehensive product characterization.

## Figures and Tables

**Table 1:** Material parameters of Mg-Nd-Zn-Zr alloy.

Sample Designation	Materials	Young’s Modulus	Poisson’s Ratio
Magnesium Alloy Rib Claw	Mg-Nd-Zn-Zr	42 GPa	0.35
synthetic rib	Polyurethane	16 GPa	0.3

**Table 2:** Mesh division details.

Sample Designation	Mesh Type	Mesh Size/mm	Mesh Count	Nodes	Sample Designation
Gen1	Tetrahedra	0.4	81,818	130,776	Gen1
Gen2	Tetrahedra	0.4	86,721	138,282	Gen2
Gen3	Tetrahedra	0.4	99,362	157,449	Gen3
synthetic rib	Tetrahedra	0.4	533,722	127,072	synthetic rib

## Data Availability

The original contributions presented in this study are included in the article. Further inquiries can be directed to the corresponding authors.
